# Baseline susceptibility to alpha-cypermethrin *in Lutzomyia longipalpis* (Lutz & Neiva, 1912) from Lapinha Cave (Brazil)

**DOI:** 10.1186/s13071-015-1076-y

**Published:** 2015-09-17

**Authors:** Grasielle Caldas DÁvila Pessoa, Josiane Valadão Lopes, Marília Fonseca Rocha, Letícia C. Pinheiro, Aline Cristine Luiz Rosa, Érika Monteiro Michalsky, Edelberto Santos Dias

**Affiliations:** Laboratório de Referência em Triatomíneos e Epidemiologia da Doença de Chagas, Centro de Pesquisas René Rachou, Fundação Oswaldo Cruz, Belo Horizonte, MG Brazil; Laboratório de Leishmanioses, Centro de Pesquisas René Rachou, Fundação Oswaldo Cruz, Av. Augusto de Lima 1715, Barro Preto, 29190-002 Belo Horizonte, MG Brazil; Centro de Controle de Zoonoses de Montes Claros, Montes Claros, MG Brazil

**Keywords:** *Lutzomyia longipalpis*, Insecticide resistance, Alpha-cypermethrin, Susceptibility reference lineage

## Abstract

**Background:**

Given the increase in cases of visceral leishmaniasis in recent years, associated with the socio-economic impact of this disease, as well as the wide distribution of *Lutzomyia longipalpis* in Brazil and the likelihood that this vector may develop resistance to insecticides used for control, the Ministry of Health considers as crucial the creation of a network in order to study and monitor the resistance of this vector to insecticides used for control. In this sense, this study aimed: 1) to characterize the susceptibility of *L. longipalpis* from Lapinha Cave (Lagoa Santa, MG - Brazil) to Alfateck SC200 in field bioassays, and 2) to define the susceptibility baseline to alpha-cypermethrin in laboratory bioassays, checking the possibility of using it as susceptibility reference lineage (SRL).

**Findings:**

The field bioassays revealed that the tested population was highly susceptible to alpha-cypermethrin in all time periods with high mortality (~100 %) in all treated surfaces before six months after spraying. In the laboratory bioassays, the studied population presented LD_50_, LD_95_ and LD_99_ to 0.78013, 10.5580 and 31.067 mg/m^2^, respectively. The slope was 1.454121.

**Conclusions:**

The studied population of *L. longipalpis* was considered as adequate for SRL according criterion recommended by Pan-American Health Organization and has proven susceptibility to tested insecticide in the field. One cannot rule out the possibility of finding populations of *L. longipalpis* more susceptible to alpha-cypermethrin; therefore, further research is necessary on other populations with potential use as a SRL.

## Findings

*Lutzomyia longipalpis* (Diptera, Psychodidae) has a broad distribution in Brazil and has been recorded in North, Central and South America, from Mexico to Argentina [1]. This phlebotomine sand fly is considered a vector for visceral leishmaniasis (VL), caused by *Leishmania* (*Leishmania*) *infantum*. During the urbanization of visceral leishmaniasis, from 1980 to 2005, 59,129 new cases of VL have been reported in Brazil [2]. In 2013, 3117 new cases of VL were reported in the whole national territory [3].

There is increasing evidence for the importance of vector control against leishmaniasis, especially for reducing risks of transmission of VL [4–7]; recommended measures include spraying households with pyrethroid insecticides [8]. However, these efforts have not been able to prevent the geographical expansion and the rise in the incidence and lethality of VL in Brazil. Furthermore, although most sand fly species remain susceptible to all of the major classes of insecticides in the world, there is increasing evidence that some phlebotomine sand flies may be developing insecticide resistance [9]. In many vector species such as Neotropical phlebotomine sand flies, the occurrence of insecticide resistance is still poorly studied. Insecticide resistance has not yet been demonstrated in *L. longipalpis* but there are some indications of its occurrence in this specie [10, 11].

Therefore, considering the increase in VL cases in recent years in Brazil and the consequent socio-economic impact caused by this disease, the wide distribution of *L. longipalpis* in the country and the likelihood that this vector may develop resistance to insecticides used for control, the Ministry of Health considers as crucial the creation of a network in order to study and monitor the resistance of this vector to insecticides [12]. In response to this need, this study aimed to characterize the susceptibility of *L. longipalpis* from Lapinha Cave (Lagoa Santa, MG - Brazil) within a timeline, to the insecticide currently being used for control of phlebotomine sand flies in the country through field bioassays, as well as define the susceptibility baseline of alpha-cypermethrin in laboratory bioassays; checking the possibility of using it as a susceptibility reference lineage (SRL).

The phlebotomine sand flies were collected from Lapinha cave (licence n° SISBIO 18728–1). HP traps [13] were used for overnight indoor insect collection. After collection, the insects were allowed to settle for a few hours for conditioning during which time they were supplied with 10 % sucrose solution. In this sense, we identify 30 % of the specimens per capture, as an internal control. The species identifications adopted in our laboratory takes into account the taxonomic keys available in Young and Ducan [1], as well as comparison with specimens deposited at the Phlebotomine Sand fly Reference Collection at CPqRR/Fiocruz.

Field bioassays were carried out in Montes Claros, MG. The residents voluntarily signed a Statement of Informed Consent. A sample was formed with three domestic units (DUs), comparable in terms of type of surface (adobe with plaster, adobe without plaster, wood) and environmental characteristics, treated with alpha-cypermethrin 20 % -Alfatek ® SC’(Rogama, Brazil). This is the insecticide currently used by the Ministry of Health of Brazil for phlebotomine sand fly control, with residual effect expected for up to 6 months. Spraying was carried out by agents of endemic diseases in the region, as recommended by the Ministry of Health. A Guarani hand sprayer was used attached to a Tee-jet 8002 nozzle. Testing was performed from July to December, 2014. At 1, 30, 60, 90, 120 and 150 days after spraying the DUs, 30 females (fasting; parental generation) were submitted to wall surface tests in plastic cones for 1 h. Then, the phlebotomine sand flies were transferred to insecticide-free bottles and kept in an insectary with temperature and humidity controlled (25 ºC ± 1 ºC, 60 % ± 10 % RU). Three cones were exposed inside the intradomicile and another three cones around in the peridomicile, of each surface type, per treated DU (experimental DUs – number 1,2,3). The insects of the control group were exposed to insecticide-free surfaces of the intradomicile and peridomicile (control DU – number 4). The mortality was recovered 24 h after phlebotomine sand flies exposition. A specimen was classified as dead if it was immobile or unable to stand or fly in a coordinated way [14].

In the laboratory, the insects were subjected to impregnated paper with ten different alpha-cypermethrin (BASF, Brazil) concentrations (0.1 to7.5 mg i.a./m^2^), for one hour. For control tests, insects were exposed to impregnated paper prepared only with vaseline oil and chloroform [15]. At least three replicates of 30 females were tested per concentration. The mortality was recovered 24 h after insects’ exposition. A specimen was classified as dead if it was immobile or unable to stand or fly in a coordinated way [13]. Data from dose–response tests were analyzed using the PROBIT program [16]. The slope and the lethal doses required to kill 50 % (LD_50_), 95 % (LD_95_) and 99 % (LD_99_) of treated individuals were estimated. During the test, the temperature and humidity were controlled (25 ºC ± 1 °C , 60 % ± 10 % RU).

Results obtained in the field bioassays revealed that the tested population was highly susceptible to alpha-cypermethrin during all the time period. The mortality was close to 100 % during the six months (Table 1). The comparison of mortality observed in control and experimental DUs by the Kruskal-Wallis test revealed a significant difference between at least two of the four DUs (*p* = 2.2 × 10^−16^). The Mann–Whitney post-test showed a difference only between the control DU and experimental DUs, proving that the mortality of phlebotomine specimen was related to the presence of the insecticide on the timeline, in the intradomicile and peridomicile, in all types of surface tested. There was no statistical difference in the mortality rate observed in the intradomicile and peridomicile between different surfaces and between different experimental DUs.

**Table 1:** Mortality of *L. longipalpis* in response to exposure on different surfaces impregnated with alpha-cypermethrin 20 %

Domestic	Surfaces	1 day after spraying				30 days after spraying				60 days after spraying				90 days after spraying				120 days after spraying				150 days after spraying			
Unit (DU)	Treated	Intradomicile		Peridomicile		Intradomicile		Peridomicile		Intradomicile		Peridomicile		Intradomicile		Peridomicile		Intradomicile		Peridomicile		Intradomicile		Peridomicile	
		Tested	Dead (%)	Tested	Dead (%)	Tested	Dead (%)	Tested	Dead (%)	Tested	Dead (%)	Tested	Dead (%)	Tested	Dead (%)	Tested	Dead (%)	Tested	Dead (%)	Tested	Dead (%)	Tested	Dead (%)	Tested	Dead (%)
DU 1^a^	Wood	30	28 (93)	30	23 (77)	30	30 (100)	30	30 (100)	30	30 (100)	30	30 (100)	30	30 (100)	30	29 (97)	30	30 (100)	30	29 (97)	30	30 (100)	30	29 (97)
	Adobe with plaster	30	30 (100)	30	30 (100)	30	30 (100)	30	30 (100)	30	30 (100)	30	30 (100)	30	30 (100)	30	28 (93)	30	30 (100)	30	27 (90)	30	30 (100)	30	29 (97)
	Adobe without plaster	30	30 (100)	30	29 (97)	30	30 (100)	30	30 (100)	30	30 (100)	30	27 (90)	30	28 (93)	30	25 (83)	30	30 (100)	30	29 (97)	30	30 (100)	30	30 (100)
	Wood	30	29 (97)	30	29 (97)	30	30 (100)	30	30 (100)	30	30 (100)	30	30 (100)	30	29 (97)	30	29 (97)	30	29 (97)	30	30 (100)	30	30 (100)	30	28 (93)
DU 2^a^	Adobe with plaster	30	30 (100)	30	30 (100)	30	30 (100)	30	30 (100)	30	30 (100)	30	30 (100)	30	30 (100)	30	30 (100)	30	30 (100)	30	28 (93)	30	30 (100)	30	30 (100)
	Adobe without plaster	30	30 (100)	30	30 (100)	30	29 (97)	30	27 (90)	30	28 (93)	30	30 (100)	30	29 (97)	30	29 (97)	30	30 (100)	30	10 (33)	30	28 (93)	30	28 (93)
	Wood	30	30 (100)	30	30 (100)	30	30 (100)	30	30 (100)	30	30 (100)	30	30 (100)	30	30 (100)	30	29 (97)	30	30 (100)	30	29 (97)	30	30 (100)	30	29 (97)
DU 3^a^	Adobe with plaster	30	30 (100)	30	30 (100)	30	30 (100)	30	30 (100)	30	30 (100)	30	30 (100)	30	30 (100)	30	29 (97)	30	29 (97)	30	29 (97)	30	30 (100)	30	30 (100)
	Adobe without plaster	30	30 (100)	30	30 (100)	30	30 (100)	30	25 (83)	30	30 (100)	30	27 (90)	30	29 (97)	30	30 (100)	30	30 (100)	30	30 (100)	30	30 (100)	30	30 (100)
DU 4^b^	Wood	30	1 (3)	30	0 (0)	30	0 (0)	30	0 (0)	30	0 (0)	30	0 (0)	30	0 (0)	30	0 (0)	30	0 (0)	30	0 (0)	30	0 (0)	30	0 (0)
	Adobe with plaster	30	0 (0)	30	0 (0)	30	0 (0)	30	0 (0)	30	0 (0)	30	0 (0)	30	0 (0)	30	0 (0)	30	0 (0)	30	0 (0)	30	0 (0)	30	0 (0)
	Adobe without plaster	30	0 (0)	30	0 (0)	30	0 (0)	30	0 (0)	30	0 (0)	30	0 (0)	30	0 (0)	30	0 (0)	30	0 (0)	30	1 (3)	30	0 (0)	30	0 (0)

Comparison of mortality in the four domestic unit ; p = 2.2 × 10^−16^, Kruskal-Wallis test; p(DU1, DU2) = 0.77, p(DU 1, DU 3) = 0.45, p(DU 2, DU 3) = 0.32, p(DU 1, DU 4) = 4.8 × 10^-16,^ p(DU 2, DU 4) = 4.9 × 10^-16,^ p(DU3, DU 4) = 2.8 × 10^−16^, Kruskal-Wallis post-hoc tests (Mann–Whitney tests considering correction for multiple tests)

^a^Experimental group

^b^Control group

The choice of the susceptibility reference lineage is a critical and fundamental step that precedes bioassays that check insect resistance to insecticides. The Pan-American Health Organization (PAHO) [17] defined a SRL as a strain with more than five generations in the laboratory (without contact with insecticides and inclusion of external material), and/or one collected from an area that has never been treated with insecticides. The phlebotomine sand flies from Lapinha Cave have met this criterion, because this region has had little anthropic interference; thus, they had not been subjected to chemical control. The high susceptibility of this population of *L. longipalpis* to alpha-cypermethrin was demonstrated in field bioassays in which high mortality was observed throughout the monitoring period.

In the laboratory bioassays, the studied population presented LD_50_, LD_95_ and LD_99_ to 0.78013, 10.5580 and 31.067 mg/m^2^, respectively. The slope was 1.454121. Such data is unprecedented in the literature and lethal doses were defined for the first time in studies with phlebotomine sand flies. The LDs defined for the SRL have a direct and immediate use if this population is used as SRL for studies with *L. longipalpis*. The LD_99_ of SRL is fundamental for defining a diagnostic dose (DD) - a tool that allows the distinction of the field population vigor against the SRL, killing the most susceptible specimens and the minimally resistant specimens. In studies with triatomines, the WHO [18] recommends the use of DD = 1×LD_99_ and, for mosquitoes, 2×LD_99_ [15]. Because research on phlebotomine sand flies is still incipient, further studies are required in order to adapt this methodology to biological/physiological characteristics of *L. longipalpis*, thus defining cutoff points that are used to foster understanding of susceptibility and resistance to insecticides.

The other LDs characterized for SRL (e.g. LD_50_ and LD_95_) may be used in the initial design of the dose–response bioassays to study field populations of *L. longipalpis*. In addition, knowledge of the LD_50_ for SRL is fundamental for calculation of the resistance ratio of 50 % (RR_50_). This indicator, calculated by dividing the LD_50_ of each field population by the LD_50_ value of the SRL, allows the susceptibility status classification of the study field population. Based on this classification, actions can be taken for vector control strategies in the field. According to PAHO [17] Triatominae populations with RR >5 are considered resistant to the insecticide tested, whereas populations of *Aedes aegypti* with RR >3 are considered resistant [19]. For phlebotomine sand flies, this information does not exist, which justifies the need for a screening of various populations of *L. longipalpis* in the laboratory and in the field bioassays, simultaneously, to define such cut-off points.

The population from Lapinha Cave has been characterized as one of the putative sibling species of *L. longipalpis* [20], and it was considered as appropriate as a SRL for laboratory bioassays because: 1) it meets the criteria for SRL, recommended by PAHO and 2) it shows insecticide susceptibility in the field on the timeline confirmed in laboratory bioassays. However, one cannot rule out the possibility of finding more susceptible populations of *L. longipalpis*. This has already been verified with triatomine studies [21]. This fact reinforces the need for further research on other populations and putative sibling species of *L. longipalpis* with potential to be used as SRL, such as those from Jacobina (Bahia, Brazil) and Espírito Santo do Pinhal (São Paulo, Brazil) [12, 20].
